# TASK1 and TASK3 Are Coexpressed With ASIC1 in the Ventrolateral Medulla and Contribute to Central Chemoreception in Rats

**DOI:** 10.3389/fncel.2018.00285

**Published:** 2018-08-29

**Authors:** Xia Wang, Ruijuan Guan, Xiaomei Zhao, Danian Zhu, Nana Song, Linlin Shen

**Affiliations:** ^1^Department of Physiology and Pathophysiology, School of Basic Medical Sciences, Fudan University, Shanghai, China; ^2^Division of Nephrology, Zhongshan Hospital, Fudan University, Shanghai, China; ^3^Shanghai Key Laboratory of Medical Imaging Computing and Computer-Assisted Intervention, Fudan University, Shanghai, China

**Keywords:** TASK1, TASK3, ventrolateral medulla, pH-sensitive, chemoreception

## Abstract

The ventrolateral medulla (VLM), including the lateral paragigantocellular nucleus (LPGi) and rostral VLM (RVLM), is commonly considered to be a chemosensitive region. However, the specific mechanism of chemoreception in the VLM remains elusive. Acid-sensing ion channels (ASICs), a family of voltage-independent proton-gated cation channels, can be activated by an external pH decrease to cause Na^+^ entry and induce neuronal excitability. TWIK-related acid-sensitive potassium channels (TASKs) are members of another group of pH-sensitive channels; in contrast to AISICs, they can be stimulated by pH increases and are inhibited by pH decreases in the physiological range. Our previous study demonstrated that ASICs take part in chemoreception. The aims of this study are to explore whether TASKs participate in the acid sensitivity of neurons in the VLM, thereby cooperating with ASICs. Our research demonstrated that TASKs, including TASK1 and TASK3, are colocalized with ASIC1 in VLM neurons. Blocking TASKs by microinjection of the non-selective TASK antagonist bupivacaine (BUP), specific TASK1 antagonist anandamide (AEA) or specific TASK3 antagonist ruthenium red (RR) into the VLM increased the integrated phrenic nerve discharge (iPND), shortened the inspiratory time (Ti) and enhanced the respiratory drive (iPND/Ti). In addition, microinjection of artificial cerebrospinal fluid (ACSF) at a pH of 7.0 or 6.5 prolonged Ti, increased iPND and enhanced respiratory drive, which were inhibited by the ASIC antagonist amiloride (AMI). By contrast, microinjection of alkaline ACSF decreased iPND and respiratory drive, which were inhibited by AEA. Taken together, our data suggest that TASK1 and TASK3 are coexpressed with ASIC1 in the VLM. Moreover, TASK1 and TASK3 contribute to the central regulation of breathing by coordinating with each other to perceive local pH changes; these results indicate a novel chemosensitive mechanism of the VLM.

## Introduction

Central chemoreceptors sense changes of H^+^ concentration ([H^+^]) in cerebrospinal fluid (CSF), play an important role in respiratory regulation and contribute to acid-base homeostasis. It is generally considered that chemoreceptors in the central nervous system (CNS) mainly detect CO_2_, while carotid bodies (peripheral chemoreceptors) detect PCO_2_ in the blood (Goridis and Brunet, 2010). CO_2_ can be dissolved in CSF and penetrates membranes readily to generate H_2_CO_3_, which decomposes to H^+^ and HCO_3_^−^. Therefore, the physiological stimulation of the central chemoreceptor is H^+^ in the CSF and local extracellular fluid. It has been reported that chemoreceptors exist in many brain areas including raphe, retrotrapezoid nucleus (RTN), ventrolateral medulla (VLM), locus coeruleus (LC) and the nucleus of tractus solitaries (NTS). Among these chemosensitive areas, VLM is considered to specialize in central chemoreception (Millhorn and Eldridge, 1986). However, the mechanism of chemoreception by VLM neurons remains elusive. pH-sensitive ion channels, including acid-sensing ion channels (ASICs) and TWIK-related acid-sensitive potassium channels (TASKs), establish a new paradigm to study the chemosensory mechanism of the CNS. Molecular analysis has shown that ASICs contribute to the capacity of afferent neurons to monitor acidosis (Holzer, 2009). Our previous study also found that ASIC1 in the VLM contributes to chemoreception and the regulation of respiration (Song et al., 2016). However, it is unclear whether TASKs participate in central chemoreception.

TASKs are characterized by “leak” K^+^ currents and play key roles in maintaining the rest membrane potential by adjusting the action potential duration and modulating the response to synaptic inputs (Honoré, 2007). TASKs are also pH-sensitive, which can be by inhibited by acidification and activated by alkalization. Among the three TASK subunits, TASK1 and TASK3 are widely expressed throughout the brain, including in the VLM and raphe nuclei (Washburn et al., 2002, 2003). These subunits are sensitive to extracellular protons with different sensitivities and contribute to the regulation of neuronal excitability (Bayliss et al., 2015). The pK value of TASK1 ranges from pH 7.3 to 7.5, and that of TASK3 ranges from pH 6.5 to 6.7 (Duprat et al., 2007). Their pH sensitivities are used to differentiate TASK1 from TASK3 (Hartness et al., 2001; Czirják and Enyedi, 2002; Washburn et al., 2003). TASKs in serotonergic raphe neurons exert pH and anesthetic sensitivity *in vitro* (Washburn et al., 2002). Inhibition of the TASKs by extracellular acidosis leads to an increased excitability of brainstem respiratory neurons (Duprat et al., 2007). Extracellular alkalization decreases the excitability of neurons expressing TASKs (Berg et al., 2004). In addition, they play a major functional role in the respiratory rhythm generation of the pre-Bötzinger complex (Koizumi et al., 2010). TASK1 and TASK3 appear to serve specific and distinct roles in chemoreception and respiratory control (Buehler et al., 2017).

It seems that both TASKs and ASICs are involved in the pH sensitivity of chemosensitive neurons in the CNS. However, the colocalization of TASKs and ASICs in chemosensitive neurons has not been addressed, and little is known about the cooperation of these two types of channels in respiratory regulation. In the current study, we hypothesized that TASKs and ASIC1 are coexpressed in the VLM and cooperate in the central control of respiration. In our present study, we found both ASIC1 and TASKs (1 and 3) expressed in the VLM of rats. We then investigated the role of ASIC1 and TASKs (1 and 3) in chemoreception. Our data showed that ASIC1 and TASKs (1 and 3) were colocalized in VLM neurons. The microinjection of different TASKs blockers, including a non-selective antagonist bupivacaine (BUP), a specific TASK1 antagonist anandamide (AEA) and a specific TASK3 antagonist ruthenium red (RR), into the VLM facilitated phrenic nerve discharge (PND). In addition, the microinjection of artificial CSF (ACSF) at a pH of 7.0 or 6.5 increased integrated PND (iPND), Inspiratory time (Ti) and respiratory drive, which were inhibited by the ASIC antagonist, amiloride (AMI). Contrarily, the microinjection of alkaline ACSF decreased iPND and respiratory drive, and this effect was attenuated by AEA. Our research indicated that TASKs and ASICs contribute to the central regulation of breathing by coordinating with each other to cause the perception of local pH changes. This investigation will help to establish a new understanding of the pH-sensing mechanism of chemosensitive neurons in the VLM.

## Materials and Methods

### Animals

Male Sprague–Dawley rats (250–350 g, aged 3–4 months) were obtained from Shanghai Jiesijie Experimental Animal Co. Ltd. (Shanghai, China). All animals were kept in a room under a 12-h light-dark cycle, an ambient temperature of 22 ± 0.5°C and a relative humidity of 60 ± 2%. Food and water were given freely. The animal experiments were conducted in strict accordance with the US National Institutes of Health Guidelines for the Care and Use of Laboratory Animals and were approved by the Ethics Committee of Experimental Research, Shanghai Medical College, Fudan University. A total of 58 adult rats were used in this study. Maximal efforts were undertaken to minimize the number of animals and their suffering.

### Drug Application

A nonselective ASIC inhibitor, AMI (Sigma Aldrich, St. Louis, MO, USA); a non-selective TASK antagonist, BUP (Sigma Aldrich, St. Louis, MO, USA); and a specific TASK3 antagonist, RR (Sigma Aldrich, St. Louis, MO, USA) were freshly prepared in ACSF immediately before administration. A specific TASK1 antagonist, AEA (Sigma Aldrich, St. Louis, MO, USA), was prepared in ethanol. The ACSF solutions were prepared at different pHs (8.0, 7.4, 7.0, 6.5, and 6.0). ACSF containing (mM) NaCl 130, NaHCO_3_ 26, KCl 5, CaCl_2_ 2.6, MgSO_4_ 1.2, NaH_2_PO_4_ 1.6, glucose 11 and sucrose 10 at pH 7.4 and ethanol served as the vehicle and volume controls.

### Immunohistochemistry

Adult Sprague-Dawley rats were anesthetized with urethane (1 g.kg^−1^) and perfused through the left ventricle with normal saline followed by 4% paraformaldehyde. After perfusion, the medullary was dissected, transferred to graded sucrose solutions (20% and 30%) until sinking and were cut into coronal sections at 25-μm thickness using a Leica freezing microtome. The slides were washed with 0.01 M PBS and blocked in 1% BSA for 1 h at room temperature. After blocking, the slides were incubated with a primary antibody against TASK1 or TASK3 (Alomone Laboratory, Israel, 1:100) diluted in 0.01 M PBS, and the controls were incubated with 0.01 M PBS without primary antibody overnight. The reaction was then detected using a Boshide avidin-biotin-HRP complex (ABC) immunohistochemical kit (Wuhan, China). Slices were dried in the drying oven and mounted with coverslips after dehydration and rendering transparent and were then observed and photographed under a microscope.

### Immunofluorescence Technique

Slides were washed with 0.01 M PBS and then blocked with a 5% mixture of donkey and goat serum for 1 h at room temperature. After blocking, the slides were incubated with primary antibodies against TASK1 (Alomone Laboratory, Israel, 1:100), TASK3 (Alomone Laboratory, Israel, 1:100) and ASIC1 (Santa Cruz Biotechnology, Dallas, TX, USA, 1:100), Neurofilament-H (Abcam, Cambridge, MA, USA) which were diluted in 0.01 M PBS, overnight. After washing, the slides were incubated with goat anti rabbit IgG conjugated with cy3, donkey anti goat IgG conjugated with FITC, rabbit anti mouse IgG conjugated with DY light 405 (1:100, Beyotime Institute of Biotechnology, Haimen, China) for 1 h in the dark. The slides were then mounted in antifading medium (Beyotime Institute of Biotechnology), and fluorescence was detected using a Zeiss LSM confocal laser system.

### Phrenic Nerve Discharge Recording

PND was recorded with platinum bipolar electrodes, which were amplified (filters set at 5.0 kHz) using a Polygraph System (NIHON KOHDEN) and digitized using a SMUP system (SMUP-E, Shanghai Medical College, Fudan University). The experiments were started after the phrenic activity was stabilized (approximately 30 min). The iPND was obtained as a moving average of the phrenic signal. Ti was averaged over 30 s. The value of each iPND and the period of Ti reflected the respiratory drive.

### Microinjection

Rats were held in stereotaxic frames with their heads inclined forward at 45 degrees to the level of the dorsal surface of the brain stem after anesthetization. A stainless steel needle was used to unilaterally microinject 0.1 μL into the VLM (12.3 mm posterior, 2.2 mm lateral, and 10 mm dorsal from the bregma). At the end of the experiment, 2% pontamine sky blue was microinjected into the same injection point. The brains were then removed and fixed in 10% formalin solution. After 48 h, the brain stem was coronally sectioned (30 μm) and stained with neutral red to determine the injection site. Data was discarded, if injection site was out of VLM.

### Statistical Analysis

Data are expressed as the means ± SD. The significance of differences among the groups was evaluated using the Student *t*-test or a one-way ANOVA test. A value of *p* < 0.05 was considered statistically significant.

## Results

### Distribution of TASK1 and TASK3 in Rat VLM

**Figure 1:**
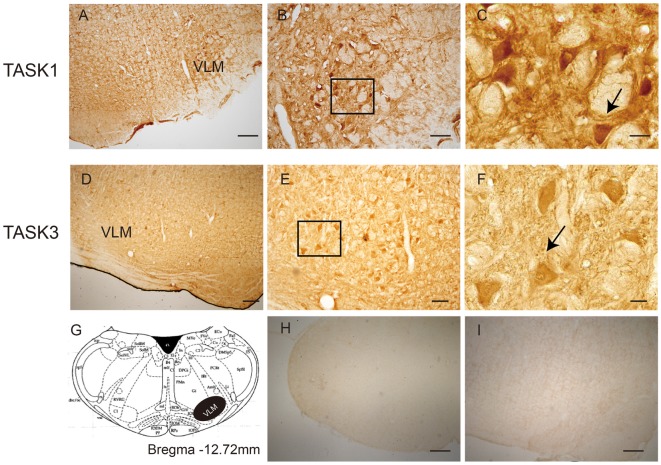
The location of TWIK-related acid-sensitive potassium channel 1 and 3 (TASK1 and 3)-positive cells in the ventrolateral medulla (VLM) of SD rats. **(A)** TASK1-positive cells were located in the VLM of rats. **(B,C)** represent high-power visual fields of the area shown in **(A)**, showing a detailed view of TASK1-positive cells in the VLM. **(D)** TASK3-positive cells in the VLM of rats. **(E,F)** represent high-power visual fields of the area shown in **(D)**, showing a detailed view of TASK3-positive cells in the VLM. **(G)** Coronal diagram of the rat medulla; the area shown in black represents the distribution of TASK1 and TASK3. Cell morphology is indicated by the arrows shown in **(C,F)**. **(H–I)** Negative control. Scale bar: 200 μm **(A,D,H,I)**; 80 μm **(B,E)** and 20 μm **(C,F)**.

The localization of TASK1 and TASK3 immunopositive cells was determined in the VLM of rats (Figure 1). According to rat brain atlases, TASK1-ir and TASK3-ir cells are mainly localized in the VLM, including the rostroventrolateral reticular nucleus (RVL) and the lateral paragigantocellular nucleus (LPGi).

### Colocalization of TASK1 and TASK3 in VLM Neurons

**Figure 2:**
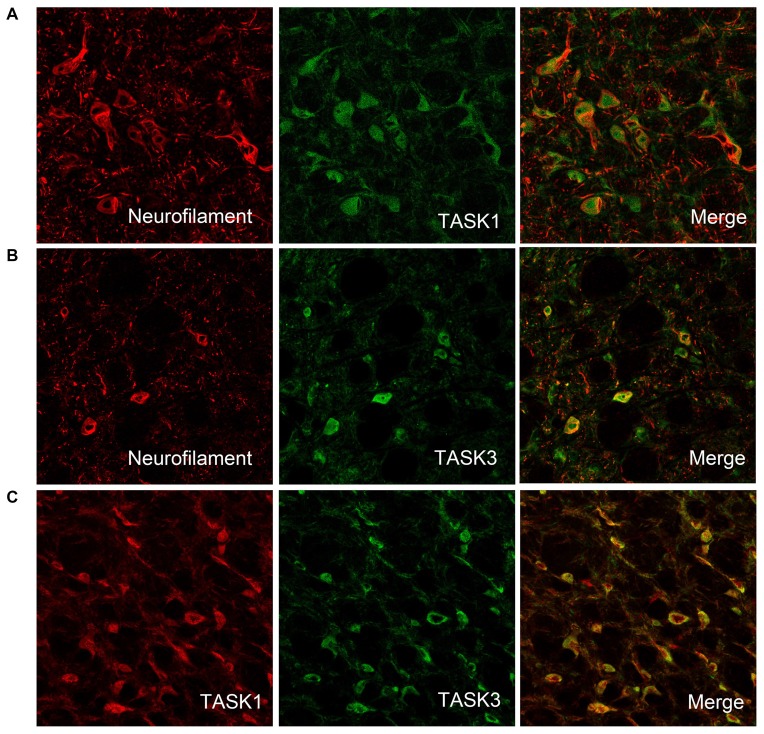
TASK1 and TASK3 are expressed and colocalized in VLM neurons. **(A,B)** Representative confocal photomicrograph showing the colocalization of TASK1-ir and TASK3-ir (green) with neurofilament-ir (red) in the VLM. **(C)** Representative confocal photomicrograph showing the colocalization of TASK1 (red) and TASK3 (green) in the VLM. Original magnification, 200×.

To detect whether TASK1 and TASK3 are localized in VLM neurons, TASK1-ir and TASK3-ir cells were measured in rat VLM by double immunofluorescence. TASK1 and TASK3 were colocalized with neurofilaments, the biomarker for neurons. Furthermore, TASK1 and TASK3 were colocalized in VLM (Figure 2).

### Coexpression of ASIC1 and TASK1 or TASK3 in VLM Neurons

**Figure 3:**
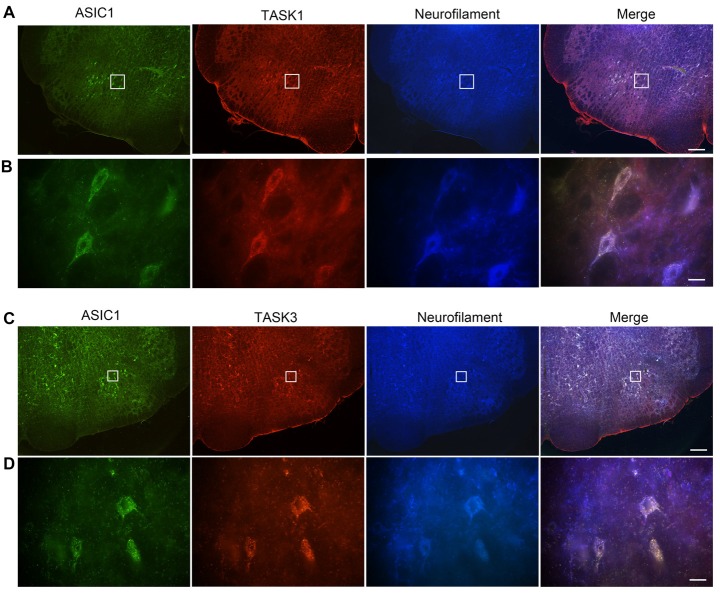
Acid-sensing ion channel 1 (ASIC1), TASK1 and TASK3 are expressed and colocalized in the VLM neurons of adult rats. **(A)** Representative confocal photomicrographs showing the colocalization of ASIC1-ir (green), TASK1-ir (red) and neurofilament-H (blue) in the VLM. **(B)** Representative high power visual field of the area shown in **(A)**. **(C)** Representative confocal photomicrographs showing the colocalization of ASIC1-ir (green), TASK3-ir (red) and neurofilament-H (blue) in the VLM. **(D)** Representative high power visual field of the area shown in **(C)**. **(A–C)** Scale bar = 200 μm; **(B,D)** scale bar = 20 μm.

We have previously shown that ASIC1 is expressed in VLM neurons and contribute to respiratory regulation (Song et al., 2016). Thus, we wondered whether ASIC1 is coexpressed with TASK1 or TASK3 in VLM neurons. Immunofluorescence was applied to observe the coexpression of ASIC1 and TASK1 or TASK3. Our data showed that ASIC1 was coexpressed with TASK1 and with TASK3 in VLM neurons (Figure 3).

### The Effects of Non-selective TASK Antagonists on Respiration

**Figure 4:**
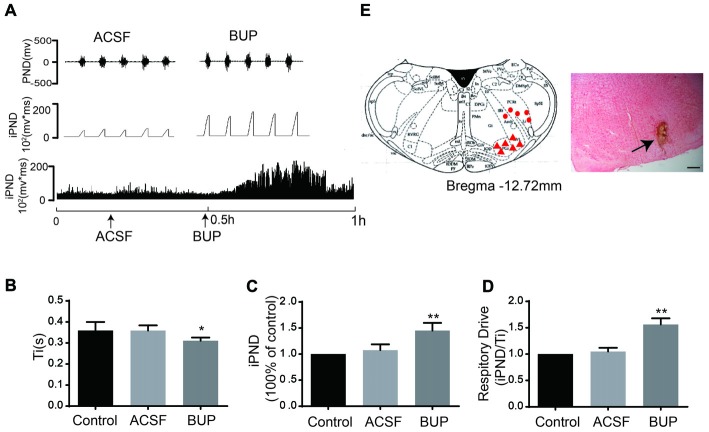
The non-selective TASKs antagonist, bupivacaine (BUP) stimulated respiration of rats. **(A)** The phrenic nerve discharge (PND) was recorded from the same animal. The unilateral microinjection of 200 μM BUP into the VLM increased PND and integrated PND (iPND). The microinjection of artificial cerebrospinal fluid (ACSF; pH 7.4) served as a control. **(B)** Inspiratory time (Ti) was decreased by BUP injection. **(C)** Group data showing the effects of BUP on iPND. **(D)** Responses of the respiratory drive. Note that BUP stimulated respiration (*p < 0.05, **p < 0.01 relative to control; *n* = 6). **(E)** Histological staining with neutral red: the sky blue spot indicates the injection site in the VLM, and the injection plot was confirmed by comparison with the Bregma −12.72 mm coronal diagram in the Paxinos and Watson stereotaxic atlas of the rat brain. In the diagram, red arrowheads represent the injection points and dots signify injection points out of VLM.

To test whether TASKs in the VLM are involved in respiration that is controlled by the respiratory center, the non-selective TASK antagonist BUP was applied. BUP was known to block TASK1 and TASK3 channels (Kindler et al., 1999).We microinjected BUP (200 μM) into the VLM to observe the consequent changes of PND and iPND (Figure 4A). Microinjection of BUP triggered significant changes in iPND, Ti and respiratory drive (integrating the value of iPND/Ti). The iPND value was increased by approximately 35% from 1.08 ± 0.11 to 1.45 ± 0.15 arbitrary units (*p* < 0.01, *n* = 6, Figure 4C). The respiratory drive was also increased from 1.05 ± 0.07 to 1.56 ± 0.12 (*p* < 0.01, *n* = 6, Figure 4D). However, Ti was shortened from 0.36 ± 0.02 to 0.31 ± 0.02 s (*p* < 0.05, *n* = 6, Figure 4B). Additionally, the injection spot was confirmed by histological staining (Figure 4E). These results indicate that the inhibition of TASKs in VLM neurons leads to cell depolarization by decreasing K^+^ efflux, thus stimulating respiration.

### The Effects of TASK1 and TASK3 Selective Antagonists on Respiration

**Figure 5:**
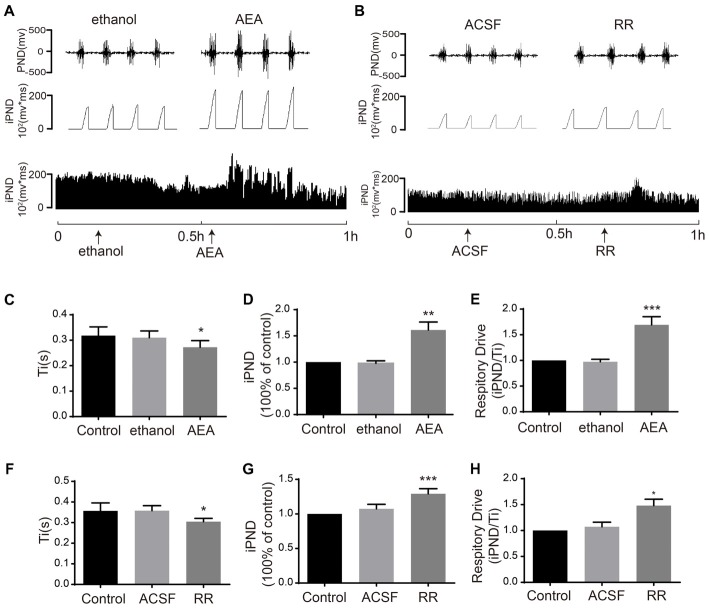
TASK1 antagonist, anandamide (AEA) and TASK3 antagonist, ruthenium red (RR) stimulated the respiration of rats. **(A,B)** The PND was recorded from the same animal. The unilateral microinjection of 100 μM AEA and 10 μM RR into the VLM increased PND and iPND. The microinjection of ethanol and ACSF (pH 7.4) served as a control. **(C–F)** Ti was decreased by AEA and RR injection. **(D–G)** Group data showing the effects of AEA and RR on iPND. **(E–H)** Responses of the respiratory drive. Note that AEA and RR stimulated respiration, *p < 0.05, **p < 0.01, ***p < 0.001 relative to control; *n* = 5.

To determine the effects of TASK1 and TASK3 on respiration, we blocked TASK1 using AEA and blocked TASK3 using RR. It was reported that AEA was widely applied as TASK1 inhibitor (Maingret et al., 2001). RR was recently found to selectively inhibit TASK3 with little or no effect on TASK-1 (Berg et al., 2004). Microinjection of AEA (100 μM) increased iPND and respiratory drive from 0.97 ± 0.04 to 1.61 ± 0.15 (*p* < 0.01, *n* = 5, Figures 5A,D) and from 0.97 ± 0.04 to 1.69 ± 0.16 (*p* < 0.001, *n* = 5, Figure 5E), respectively. Ti was shortened from 0.31 ± 0.03 to 0.27 ± 0.03 s (*p* < 0.05, *n* = 5, Figures 5C). RR (10 μM) increased iPND from 1.07 ± 0.06 to 1.29 ± 0.07 (*p* < 0.001, *n* = 5, Figures 5B,G), enhanced respiratory drive from 1.07 ± 0.09 to 1.48 ± 0.12 (*p* < 0.05, *n* = 5, Figure 5H), and shortened Ti from 0.36 ± 0.02 to 0.31 ± 0.02 s (*p* < 0.05, *n* = 5, Figure 5F). Together, these results suggest that both TASK1 and TASK3 in the VLM are involved in the central regulation of respiration. Furthermore, inhibition of TASK1 and TASK3 in rat VLM stimulated breathing that is controlled by the respiratory center.

### The Effect of ACSF With Different pH Values on Respiration

**Figure 6:**
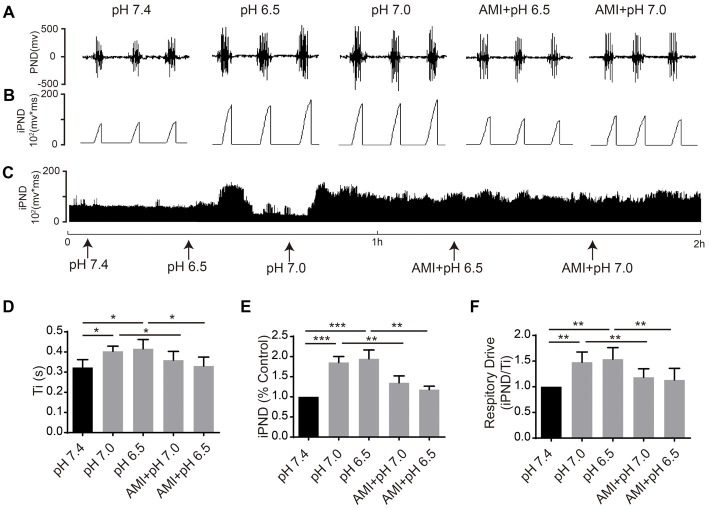
ASIC antagonists attenuated the stimulatory effect of acidification on respiration in rat VLM. **(A–C)** The unilateral microinjection of 0.1 μL ACSF (pH 7.4, 7.0 and 6.5) into the VLM increased raw PND **(A)** and iPND **(B,C)**. AMI pre-treatment (100 μM) blocked this effect. The microinjection of ACSF (pH 7.4) served as a control. **(D–F)** Statistical data showing the effects of alkalization and AMI on Ti **(D)**, iPND **(E)** and respiratory drive **(F)**. *p < 0.05, **p < 0.01, ***p < 0.001, *n* = 6.

**Figure 7:**
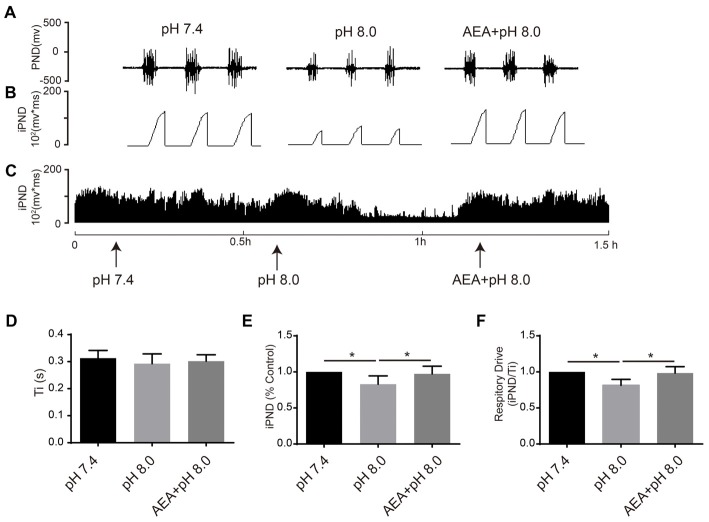
TASK1 antagonists attenuated the subdued effect of alkalization on respiration in the rat VLM. **(A–C)** The unilateral microinjection of 0.1 μL ACSF (pH 8.0) into the VLM decreased raw PND **(A)** and iPND **(B,C)**. TASK1 antagonist AEA pre-treatment (100 μM) blocked this effect. The microinjection of ACSF (pH 7.4) served as a control. **(D–F)** Statistical data showing the effects of different levels of alkalization and AEA on Ti **(D)**, iPND **(E)** and respiratory drive **(F)**. *p < 0.05, *n* = 6.

It has been reported that TASKs are sensitive to changes in extracellular proton chemoreception and that the inhibition of TASKs by extracellular acidosis leads to increased excitability of brainstem respiratory neurons (Duprat et al., 1997; Bayliss and Barrett, 2008). Conversely, activation by alkalization exerts an inhibitory effect. ASICs have been reported to be involved in the pH sensitivity in the CNS and are voltage-insensitive, proton-gated cation channels that are activated by extracellular acidification (Waldmann et al., 1997). AMI is widely used as a non-selective ASIC inhibitor (Waldmann et al., 1997; Baron and Lingueglia, 2015). We first microinjected ACSF at acidic pH (7.4, 7.0 and 6.5) into the VLM of rats and observed the resulting changes of respiratory activation (Figures 6A–C). Microinjection at pH 7.0 and 6.5 caused significant increases in iPND, Ti and respiratory drive, which were inhibited by AMI (Figures 6D–F). To explore the effect of TASK activation on respiration, we microinjected alkaline ACSF (pH 8.0) and found that alkaline ACSF triggers decreases of iPND and respiratory drive, which were inhibited by AEA (Figures 7A,B,C,E,F). However, microinjection of alkaline ACSF had no significant effect on Ti (Figure 7D).

## Discussion

The central chemoreflex is essential to the maintenance of circulatory acid-base homeostasis by adjusting the activity of breathing. However, the mechanism of chemoreflex has remained unclear until now. Our previous study demonstrated that ASICs are expressed on the neurons of chemosensitive areas such as the lateral hypothalamus and VLM and contribute to respiratory regulation. In the present study, we focused on the role of another class of pH-sensitive ion channels, TASKs, in breathing regulation and their cooperation with ASICs in the central chemosensory system. We found that TASK1 and TASK3 are expressed in the VLM of rats. TASK1 and TASK3 cooperate with ASIC1 to participate in the central regulation of respiration. This might be the chemosensitive mechanism of the VLM.

The expression of TASKs in brain stem has been reported previously (Talley et al., 2001; Berg et al., 2004). TASK1 is expressed in the facial nucleus, ambiguous nucleus, hypoglossal nucleus and LC (Sirois et al., 2000; Bayliss et al., 2001), and TASK3 is expressed in the nucleus of the solitary tract, raphes and LC (Talley et al., 2001). ASICs, especially ASIC1 and ASIC2a, are widely expressed in the CNS (García-Añoveros et al., 1997; Waldmann et al., 1997; Alvarez de la Rosa et al., 2003). It has been reported that ASIC1 in NTS mediates chemosensitivity and is involved in the control of breathing (Huda et al., 2012). Acidification of the LH can stimulate breathing via the activation of ASIC1a on orexin neurons (Song et al., 2012). However, little is known about the coexpression of ASIC1 and TASKs (1 and 3) in the CNS. In the present study, we first examined the expression of TASK1 and TASK3 by localizing TASK1- and TASK3-positive cells in the VLM by immunohistochemistry. TASK1 and TASK3 subunits are colocalized with most serotonergic dorsal and caudal raphe neurons and with noradrenergic cells of the LC (Talley et al., 2001; Washburn et al., 2002). According to the immunofluorescence results, TASK1 and TASK3 coexpression were detected in the VLM, which indicates that heteromeric TASKs may also be involved in central chemoreception. Furthermore, ASIC1, which can be activated by extracellular acidification, was found to be coexpressed with TASK1 and TASK3, suggesting that TASKs may cooperate with ASIC1 and thereby participate in central respiratory regulation. The VLM neurons serve as central chemoreceptors mediating respiratory responses to hypoxia and/or hypercapnia (Wakai et al., 2015) Therefore, these morphological results lay the foundation for exploring the function of ASIC1 and TASKs (1 and 3) in the brainstem.

Recent studies have shown that TASK1 and TASK3 subunits generate a pH-sensitive and weakly rectifying K^+^ current, which is critical for regulating the resting membrane potential and the excitability of respiration-related neurons (Duprat et al., 1997; Buckler et al., 2000; Kim et al., 2000; Bayliss et al., 2015). TASK1 and TASK3 are responsible for the pH sensitivity of serotonergic neurons in the dorsal and caudal raphe of mice (Washburn et al., 2002). *In vivo*, hypoxic and acidic responses are partially blunted in TASK1 and TASK3 knockout mice (Trapp et al., 2008). In the carotid body, TASK1 plays a key role in the control of ventilation peripherally in mice (Trapp et al., 2008). Despite these findings, the role of TASKs in the VLM in central respiratory regulation has not been reported yet. We determined the role of TASK1 and TASK3 in the central regulation of breathing by PND recording. TASK1 and TASK3 are both inhibited by local anesthetics, including BUP (Kindler et al., 1999; Kim et al., 2000). It has been demonstrated that TASK1 is directly inhibited by the AEA (Maingret et al., 2001), and TASK3 is selectively blocked by RR (Czirják and Enyedi, 2002). Therefore, a non-selective antagonist, BUP, a specific TASK1 antagonist, AEA, and a specific TASK3 antagonist, RR, were applied in an animal experiment. We found that the microinjection of BUP, AEA and RR stimulated breathing by increasing PND and iPND, shortened Ti, and enhanced respiratory drive, suggesting that the inhibition of TASK1 and TASK3 in VLM neurons blocked the background K^+^ current, which contributes to producing the action potential and increases the excitability of inspiratory neurons. However, neither RR nor AEA are specific for TASKs channels. This is a shortcoming of the study. RR is a non-selective inhibitor of transient receptor potential (TRP) channels and AEA is agonist of both cannabinoid (CB) receptors and TRP channels (Watanabe et al., 2003; Kopczyńska, 2007). Indeed, it has been reported that CB receptors and TRP channels cooperate with each other in breathing regulation. Intravenous injection of AEA induced depression of breathing can be prevented by the CB1 antagonist AM281 (Kopczyńska, 2007; Iring et al., 2017). TRPV2 channel was found to be expressed in VLM (Nedungadi et al., 2012). However, TRPV2 channel is mainly stretch and thermo sensitive. TRPV1 is acidic sensitive, which can be activated by low pH. Our current study found that microinjection of either agonist (AEA) or antagonist (RR) of TRP channels into VLM stimulates breathing. Thus, we speculate that it is unlikely TRP take part in regulation of breathing by neurons of the VLM. Ordinarily, activation of CB receptors reduces neuronal excitability (den Boon et al., 2012). But, we found that microinjection of the agonist of CB receptors, AEA into the VLM activated breathing. Thus, we consumed that it is unlikely the activation of CB receptor medicated respiratory regulation. However, there is no direct evidence to rule out the role of CB receptors and TRP channels in VLM mediated respiratory regulation. Further investigations are needed to explore the roles of TASKs channels in the central regulation of breathing.

It has been well documented that central respiratory chemoreceptors are located in the medullary raphe, nucleus tractus solitarius, VLM and hypothalamus (Funk, 2010). VLM, including RVL and LPGi, is a putative site for central chemoreception, (Corcoran et al., 2009). However, the specific pH sensing mechanism remains controversial. Therefore, we focused on the effect of ASIC1 and TASKs in the VLM on chemoreception.

TASKs are inhibited by extracellular acidification (Bayliss et al., 2003; Bayliss and Barrett, 2008; Enyedi and Czirják, 2010). The pH sensitivities of heterodimeric TASKs (1 and 3) and TASK1 (pK ~7.4) are closer to the physiological range than that of TASK-3 (pK ~6.8; Berg et al., 2004; Duprat et al., 2007). Thus, the acid-induced inhibition of TASK channel activity enhances nerve excitability. In addition to TASKs, ASICs have been reported to be involved in the pH sensitivity in the CNS; ASICs are voltage-insensitive, proton-gated cation channels that are activated by extracellular acidification (Waldmann et al., 1997). ASIC1 activation causes the entry of Na^+^ and a little Ca^2+^, which leads to the polarization and excitability of neurons. Because of the similar pKa values of TASK3 and ASIC1a and their coexpression in the VLM, we think that these proteins may work together to participate in respiratory regulation by central chemosensitive neurons. AMI is widely used to block ASICs and can reversibly inhibit ASICs (Champigny et al., 1998; Baron and Lingueglia, 2015). To investigate whether TASKs (1 and 3) and ASIC1 contribute to chemoreception in rats, we microinjected ACSF at pH values from 7.4 to lower pH levels dose-dependently in the absence and presence of AMI into VLM to observe the change of PND. Our results showed that the microinjection of ACSF at pH 7.0 and 6.5 obviously prolonged Ti, increased iPND, and enhanced respiratory drive. AMI inhibited this effect. On the contrary, the microinjection of ACSF at pH 8.0 decreased iPND and respiratory drive, which were inhibited by AEA. However, alkaline ACSF had no significant effect on Ti. One possible reason is that TASK1 and TASK3 exhibit different pH sensitivity. TASK-1 is activated strongly by alkalization pH over 7.3 (Duprat et al., 1997; Bayliss et al., 2001, 2015), whereas, TASK3 is nearly fully activated at pH 7.3, and additional alkalization has little effect on TASK-3 currents (Rajan et al., 2000; Bayliss et al., 2015). The results indicated that activation of TASK-1 may have minor effect on Ti. However, further studies are needed to explore the roles of TASKs in regulation of breathing.

Based on the data presented above, the inhibition of TASK1 and TASK3 in the VLM under physiological conditions stimulates respiration. The acidification of the VLM led to PND enhancement, which was caused by ASIC1 activation. The alkalization of the VLM reduced PND, which was mainly caused by TASK1 activation. This result indicates that ASIC1 and TASKs (1 and 3) coexist in the chemosensitive neurons of VLM and coordinate with each other to sense local pH fluctuations; this may be one of the mechanisms by which central chemosensitive neurons respond to pH changes.

In summary, extracellular pH is a critical signal in the central regulation of breathing. In the present study, we have shown that TASKs including TASK1 and TASK3 are expressed in the VLM of rats and are coexpressed with ASIC1 in VLM neurons. Furthermore, we have shown that local acidification of the VLM can stimulate respiration, mainly via ASIC1. Local alkalization weakens respiration, mainly via TASK1. Our findings support the notion that ASIC1, TASK1 and TASK3 are expressed on neurons in the VLM and participate in the chemical regulation of respiration in response to extracellular pH change under physiological conditions.

## Author Contributions

XW performed the *in vivo* and some of the *in vitro* experiments and prepared the manuscript. RG conducted some of the *in vitro* experiments. XZ and DZ analyzed some of the data. NS and LS designed the experiments and revised the manuscript.

## Conflict of Interest Statement

The authors declare that the research was conducted in the absence of any commercial or financial relationships that could be construed as a potential conflict of interest.
